# Zika Virus Induces Autophagy in Human Umbilical Vein Endothelial Cells

**DOI:** 10.3390/v10050259

**Published:** 2018-05-15

**Authors:** Haoran Peng, Bin Liu, Toure Doueu Yves, Yanhua He, Shijie Wang, Hailin Tang, Hao Ren, Ping Zhao, Zhongtian Qi, Zhaoling Qin

**Affiliations:** Department of Microbiology, Shanghai Key Laboratory of Medical Biodefense, Second Military Medical University, Shanghai 200433, China; phran@126.com (H.P.); smmuliubin@163.com (B.L.); chabaiichabaii@gmail.com (T.D.Y.); hyh0556@163.com (Y.H.); 15026921726@163.com (S.W.); hailint@163.com (H.T.); hren2013@139.com (H.R.); pnzhao@163.com (P.Z.)

**Keywords:** Zika virus, autophagy, LC3, Beclin-1, viral replication, virus production

## Abstract

Autophagy is a common strategy for cell protection; however, some viruses can in turn adopt cellular autophagy to promote viral replication. Zika virus (ZIKV) is the pathogen that causes Zika viral disease, and it is a mosquito-borne virus. However, its pathogenesis, especially the interaction between ZIKV and target cells during the early stages of infection, is still unclear. In this study, we demonstrate that infecting human umbilical vein endothelial cells (HUVEC) with ZIKV triggers cellular autophagy. We observed both an increase in the conversion of LC3-I to LC3-II and increased accumulation of fluorescent cells with LC3 dots, which are considered to be the two key indicators of autophagy. The ratio of LC3-II/GAPDH in each group was significantly increased at different times after ZIKV infection at different MOIs, indicating that the production of lipidated LC3-II increased. Moreover, both the ratio of LC3-II/GAPDH and the expression of viral NS3 protein increased with increasing time of viral infection. The expression level of p62 decreased gradually from 12 h post-infection. Expression profile of double fluorescent protein labelling LC3 indicated that the autophagy induced by ZIKV infection was a complete process. We further investigated the role of autophagy in ZIKV replication. We demonstrated that either the treatment with inhibitors of autophagosomes formation or short hairpin RNA targeting the Beclin-1 gene, which is critical for the formation of autophagosomes, significantly reduced viral production. Taken together, our results indicate that ZIKV infection induces autophagy of HUVEC, and inhibition of ZIKV-induced autophagy restrains viral replication.

## 1. Introduction

Zika virus (ZIKV) was isolated in 1947 in Uganda [1], and it is a reemerging, mosquito-borne flavivirus. It is mainly transmitted by different *Aedes* species, such as *Aedes aegypti*. However, cases of mother-to-fetus transmission during pregnancy and sexually transmitted infections have been recently reported [2,3,4,5]. The typical symptoms of infected individuals include fever, rash, arthralgia, and conjunctivitis, but incidences of Guillain Barré syndrome and neurological disorders were also observed [6,7,8]. Despite receiving increased attention since its outbreak in 2016, the pathogenesis of ZIKV infection is still poorly understood.

ZIKV is a single-stranded positive sense RNA virus that belongs to the *Flaviviridae* family [9]. Similar to other members of the *Flavivirus* genus, ZIKV genomic RNA encodes a long polyprotein. The polyprotein is then cleaved and processed into three structural proteins (precursor membrane (prM), envelope (E) and capsid (C)) and seven nonstructural proteins (NS1, NS2A, NS2B, NS3, NS4A, NS4B, and NS5) by host or viral proteases [10]. ZIKV can replicate and establish infections in many cell types. To date, the molecular mechanisms underlying the early stages of ZIKV infection remain unclear.

Autophagy is a highly conserved catabolic process in which long-lived cytoplasmic components or damaged organelles are sequestered through the formation of double-membraned autophagosomes. Mature autophagosomes ultimately fuse with lysosomes to form single-membraned autophagolysosomes that degrade or recycle their contents [11]. Upon autophagy initiation, microtubule-associated protein 1 light chain 3 (LC3) is converted from LC3-I to the lipidated LC3-II and anchored to the autophagic membrane. The punctate distribution of LC3-II is considered a marker of autophagy induction, and it is closely related to the accumulation of autophagosomes [12]. Lipidated LC3-II usually interacts with p62, which is a multifunctional protein that is degraded by the autophagic-lysosome pathway. The occurrence of complete autophagic flux is commonly reflected in the expression of LC3-II and p62 [13]. The regulatory mechanisms mediated by phosphatidylinositol 3-kinase (PI3K), the mammalian target of rapamycin (mTOR), and autophagy-related protein Beclin-1 are confirmed to play crucial functions in the autophagy progress [13,14,15].

As an innate host defense response, autophagy can be induced by various stress stimuli, including nutrient starvation and viral infection [16]. Under these circumstances, autophagy is employed by host cells to ensure the survival of infected cells by removing damaged organelles and maintaining cellular homeostasis, as demonstrated by herpes simplex virus type-1(HSV-1) and vesicular stomatitis virus (VSV) [17,18]. However, some single-stranded RNA viruses, such as dengue virus [19], coxsackievirus, and hepatitis C virus, develop an escape mechanism that evades the monitoring and protective functions of autophagy induced by host cells [19,20,21]. Meanwhile, some viruses take advantage of autophagy instead to benefit their own replication, and this has been demonstrated by several members of the *Flavivirus* genus [22]. ZIKV infection of human fetal neural stem cells (fNSCs) was found to cause inhibition of the Akt-mTOR pathway, leading to defective neurogenesis and aberrant activation of autophagy [23]. Another group discovered that secretory autophagy may facilitate ZIKV transfer across the placental barrier, and regulations to the equilibrium between degradative autophagy and secretory autophagy may influence the incidence of microcephaly [24]. Further studies demonstrated that inhibition of autophagy limits vertical transmission of Zika virus in pregnant mice [25], which corresponded with the previous work. 

Since ZIKV can be transmitted to humans via mosquito-transmission, systemic viral infection requires ZIKV to infiltrate blood vessels and spread through the circulatory system. A previous study showed that ZIKV infection of skin fibroblasts resulted in the formation of autophagosomes [26]. Further studies are needed to explore the exact mechanism of ZIKV-induced autophagy and the functional role for viral replication. In this paper, we provide evidence that autophagy is triggered in human umbilical vein endothelial cells (HUVECs) after ZIKV infection, and inhibition of ZIKV-induce autophagy can restrain viral replication to some extent by regulating the autophagy pathway through pharmacological drugs and RNA interference.

## 2. Materials and Methods

### 2.1. Cells

Human umbilical vein endothelial cells (*HUVEC*) and Vero cells were grown at 37 °C in complete Dulbecco’s Modified Eagle Medium (DMEM) containing 10% (*v*/*v*) heat-inactivated fetal bovine serum (FBS) (Gibco BRL, Carlsbad, CA, USA) supplemented with 100 nM nonessential amino acids (NEAA) (Invitrogen, Shanghai, China), 1 mM l-glutamine, 100 μg streptomycin/mL, and 100 U penicillin/mL. C6/36 cells (kindly provided by Professor Jing An from Capital Medical University, Beijing, China) were cultured in RPMI1640 (Gibco) supplemented with 10% FBS.

### 2.2. Antibodies and Chemicals

Rapamycin and wortmannin were purchased from Selleck (Shanghai, China). Chloroquine and a polyclonal rabbit anti-LC3B antibody were purchased from Sigma Aldrich (St. Louis, MO, USA). Polyclonal rabbit anti-DENV NS3 was purchased from GeneTex (Irvine, CA, USA). Monoclonal rabbit anti-Beclin-1 antibody was purchased from Cell Signaling Technology (Danvers, MA, USA). Monoclonal mouse anti-p62 and anti-GAPDH antibodies were obtained from Abcam (Cambridge, MA, USA) and Abmart (Shanghai, China), respectively. Horseradish peroxidase (HRP)-conjugated secondary antibodies were purchased from Beyotime (Hangzhou, China). Beclin-1 specific short hairpin RNA (shRNA) targeting the sequence CCGGCCGACTTGTT-CCTTACGGAAACTCGAGTTTCCGTAAGGAACAAGTCGGTTTTTG was purchased from Sigma Aldrich (St. Louis, MO, USA).

### 2.3. Plasmid Construction

The mTagRFP-mWasabi-LC3 plasmid was a kind gift from Professor Jian Lin from Peking University, Beijing, China. The sequence encoding mTagRFP-mWasabi-LC3 was cloned and inserted into lentiviral vector using exogenously added Xba I and Sal I sites. Sequence analysis of the construct was performed by RuiDi (Shanghai, China).

### 2.4. Viral Infection

ZIKV (strain GZ01, kindly provided by Professor Cheng-Feng Qin from Beijing Institute of Microbiology and Epidemiology, Beijing, China) was propagated in C6/36 cells, and the stock virus was stored at −80 °C until use. Virus titers were detected by plaque assay and measured as the plaque-forming unit (PFU) per milliliter. HUVEC were infected with ZIKV at a multiplicity of infection (MOI) of 1 or indicated MOIs for 1 h in serum-free opti-MEM. Then, the cells were washed with PBS and cultured in the fresh complete media for various times until they were harvested and examined. For autophagy induction or inhibition experiments, cells were treated with different concentrations of rapamycin or inhibitors for 2 h prior to virus infection. The cells were infected with ZIKV at an MOI of 1 for 1 h, washed, and then incubated with fresh complete medium in the presence or absence of various drugs.

### 2.5. Lentiviral Particle Production and Target Cell Transduction

Lentiviral particles pseudotyped by vesicular stomatitis virus glycoprotein (VSV-G) were produced in 293T cells by co-transfection of lentiviral vector encoding short hairpin RNA (shRNA) against Beclin-1 (Sigma Aldrich, St. Louis, MO, USA) or mTagRFP-mWasabi-LC3 with vectors encoding compatible packaging proteins and VSV-G as previously described [27]. Forty-eight hours after transfection, cell supernatants were collected, centrifuged, filtrated, and adopted to transduce HUVEC at 50 to 80% confluence. An empty lentivirus vector was used as a negative control. The silencing efficiency was detected by western blotting using the specific anti-Beclin-1 antibody. Forty-eight hours post-transduction, the cells were infected with ZIKV at the indicated MOIs, as described above.

### 2.6. Plaque Assay

Virus titers in the culture supernatant were determined by plaque assay performed in triplicate, as previously described [28]. Briefly, a Vero cell monolayer was infected with the serially diluted supernatants from ZIKV-infected cells. After 1 h of adsorption, the cells were washed with PBS, and then overlay medium containing 1.3% methylcellulose was added to each well. Following incubation at 37 °C for 6 to 7 days, the cells were stained with 1% crystal violet staining. Plaques were counted, and the viral titers were calculated as PFU per milliliter.

### 2.7. Confocal Microscopy

For the detection of autophagosomes, cells were infected with lentivirus expressing mTagRFP-mWasabi-LC3. After 48 h of lentiviral infection, cells were infected with ZIKV for 1 h, and the recombinant fluorescent LC3 was observed under a Zeiss or Leica SP8 confocal fluorescence microscope. The number of cells with punctate fluorescent LC3 localization relative to all positive fluorescent cells was counted (a minimum of 100 fluorescence-positive cells were counted in total for each condition) and presented as a percentage, as previously described [29].

### 2.8. Western Blot Analysis

Western blot analysis was performed as previously described, with some modifications [30,31]. Briefly, cells were lysed with RIPA lysis buffer (Beyotime, China) containing protease inhibitor cocktail and 1% phenylmethylsulfonyl fluoride (PMSF) on ice. After centrifugation, protein concentrations were determined using the Bradford method (Beyotime). Proteins were then separated using 12.5% (*w*/*v*) SDS-polyacrylamide gel electrophoresis (PAGE), and transferred onto PVDF membranes (Millipore, Burlington, MA, USA) using a Trans-Blot apparatus (Bio-Rad, Hercules, CA, USA). The proteins of interest were identified specifically using primary antibodies, and detected visually using horseradish peroxidase (HRP)-conjugated species-specific secondary antibodies. Immunoreactivity was visualized by enhanced chemiluminescence technique using SuperSignal West Pico chemiluminescent substrate (Thermo, Waltham, MA, USA).

### 2.9. Statistical Analysis

Data are represented as the means ± standard deviations (SD) of independent experiments. Values of *p* < 0.05 (*), *p* < 0.01 (**), and *p* < 0.001 (***) were considered to be statistically significant using the Student’s *t*-test and Prism software.

## 3. Results

### 3.1. ZIKV Infection Induces Autophagy in HUVEC

**Figure 1:**
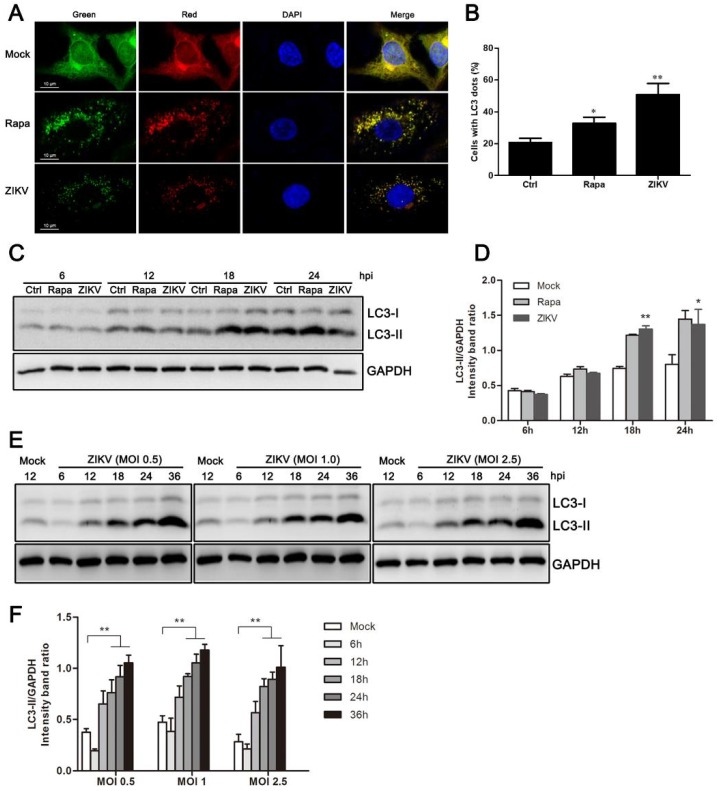
ZIKV induces autophagy in HUVEC. (**A**) Confocal microscopy. HUVEC were infected with lentivirus expressing mTagRFP-mWasabi-LC3 followed by treatment at 48 h post-infection with mock treatment as a negative control, rapamycin (100 nM) treatment as a positive control, or infection of ZIKV (MOI = 1) for 24 h. The cells were observed by confocal microscopy with scale bars indicating 10 µm; (**B**) percent of cells with LC3 dots from total LC3-expressing cells was calculated. Data were derived from at least 100 cells for each sample. * *p* < 0.05, ** *p* < 0.01; (**C**) western blot analysis. The turnover of LC3-I to LC3-II was detected for mock-treated cells, rapamycin-treated (100 nM) cells, or the infected cells with ZIKV at MOI 1. Cells were harvested at indicated time points and detected with anti-LC3B antibody. GAPDH was used as a loading control. Representative images are presented; (**D**) the intensity band ratio of LC3-II to GAPDH. Graphs represent the ratio of LC3-II to GAPDH, as measured with densitometry; (**E**) HUVEC were infected with ZIKV at a range of MOIs (0.5, 1, and 2.5) for the indicated time, and LC3 expression was measured by western blot. The ratios of LC3-II to GAPDH were calculated and presented (**F**). Data are presented as means from three independent experiments. Significance was analyzed with two-tailed Student’s t test. * *p* < 0.05, ** *p* < 0.01.

A previous study has shown that ZIKV infection induces autophagy in human fetal neural stem cells via its NS4A and NS4B proteins by deregulating Akt-mTOR signaling [23]. To determine whether the autophagic response of HUVEC is also triggered by ZIKV infection, a reporter construct named mTagRFP-mWasabi-LC3 was used. This tandem-tag reporter construct enables the detection of autophagic flux. The low-pH of lysosomes quenches the green signal and therefore puncta that are only red suggests autolysosomes, whereas puncta that appears red and green, or yellow when merged, indicate autophagosomes [32]. HUVEC were infected with lentivirus expressing mTagRFP-mWasabi-LC3 for 48 h, and were then infected with ZIKV at an MOI = 1 for 24 h. Mock or infected cells were observed under confocal microscopy to verify the activation of the autophagy machinery. Compared with mock-infected cells, the amount of fluorescent LC3 puncta noticeably increased in ZIKV-infected cells. However, a largely dispersed fluorescence distribution was observed in the cytoplasm of mock-infected cells (Figure 1A). Further quantitative analysis indicated that the number of cells with fluorescent LC3 dot formation increased during ZIKV infection. We also observed an increase in fluorescent LC3 puncta and an increase in the number of fluorescent cells with dot formation in cells treated with an autophagosome inducer, rapamycin (Figure 1B).

To confirm that the fluorescent dot formation from the morphological observations was indeed related to the induction of autophagy, LC3 modification was examined by western blot analysis. The LC3-II/GAPDH ratio was then calculated to reflect autophagy activity. The conversion of endogenous LC3-I to LC3-II was monitored at 6, 12, 18, and 24 h post-ZIKV infection at an MOI of 1. Rapamycin treatment of HUVEC increased the conversion to LC3-II, and it was used as a positive control. As demonstrated in Figure 1C, ZIKV-infected HUVEC had substantial LC3-I to LC3-II conversion in the late stages of infection. To quantify these results, band intensity was measured by densitometry analysis. The densitometry ratio of LC3-II to GAPDH was increased at 18 h and 24 h in ZIKV-infected cells when compared to the mock-infected cells (Figure 1D). Comigration of LC3-II in infected cells and in cells treated with rapamycin confirmed that the band was authentic LC3-II. Additionally, similar results were obtained in ZIKV-infected cells at MOIs of 0.5, 1.0, and 2.5 (Figure 1E). Correspondingly, we detected a significant increase in the expression of LC3-II in ZIKV-infected cells by 18 h post-infection (hpi), and it was maintained until at least 36 hpi. (Figure 1E,F). Collectively, these data demonstrate that ZIKV infection induced an autophagic response in HUVEC.

### 3.2. Increased Levels of Autophagic Flux in ZIKV-Infected HUVEC

**Figure 2:**
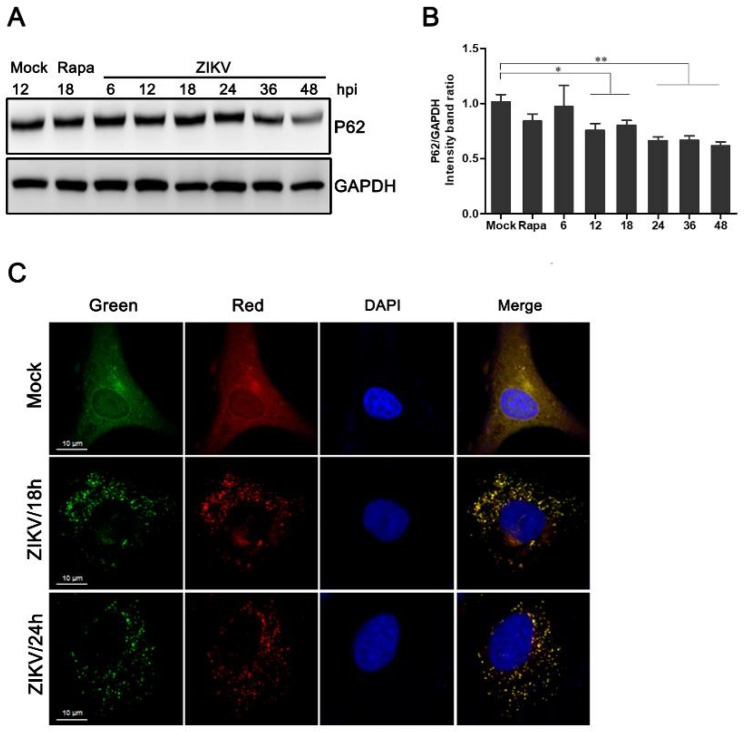
Measurement of the autophagic flux in ZIKV-infected HUVEC. (**A**) Western blot analysis of p62 degradation. The time course of expression of p62 in ZIKV-infected HUVEC at MOI 1 was investigated using anti-p62 antibody. Mock-infected HUVEC were used as negative controls, rapamycin (100 nM) treatment was used as a positive control, and GAPDH was used as a protein-loading control; (**B**) the ratios of P62 to GAPDH were calculated, and representative results are presented with graphs. Data are presented as means from three independent experiments. Compared to the control group, significance is analyzed with two-tailed Student’s *t* test. * *p* < 0.05; ** *p* < 0.01. (**C**) HUVEC transduced with lentivirus expressing mTagRFP-mWasabi-LC3 were either uninfected or infected with ZIKV at MOI 1. The cells were fixed and visualized by confocal microscopy at 18 and 24 h post-infection, respectively. Bars, 10 µm.

P62 acts as an adaptor of LC3-II and is specifically degraded by the autophagy-lysosome pathway [33]. The inhibition of autophagy is associated with the accumulation of p62. Therefore, its degradation is considered to be an indicator in the assessment of normal autophagic flux. To explore whether the autophagic response induced by ZIKV was complete, the expression of p62 was measured by western blot analysis. Compared to the mock-infected control, the degradation of p62 in HUVEC increased starting at 12 h post-ZIKV infection. We also observed a reduction in the expression of p62 in cells treated with rapamycin. Furthermore, there was a statistically significant decline in the expression levels of p62 protein at 48 hpi (Figure 2A,B).

As shown in Figure 2C, a slight shift from yellow to partially red fluorescence was observed in ZIKV-infected HUVEC expressing mTagRFP-mWasabi-LC3 from 18 to 24 h following ZIKV infection. This indicates an increase in the level of autophagic flux (Figure 2C). Taken together, these data suggest that ZIKV infection can induce a complete autophagic response in HUVEC.

### 3.3. Pharmacological Inhibition of Autophagy Reduces ZIKV Production in HUVEC

**Figure 3:**
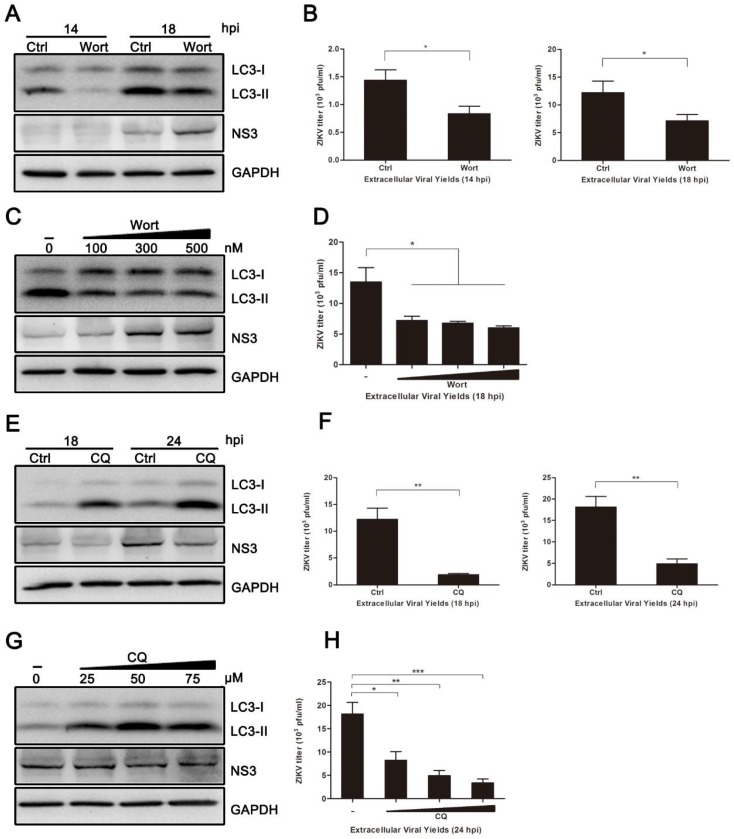
Inhibition of autophagy reduced ZIKV production. HUVEC were pretreated with wortmannin (Wort) (100 nM, or the indicated concentrations), chloroquine (CQ) (50 µM, or the indicated concentrations) or DMSO (control) in complete medium for 2 h and then infected with ZIKV (MOI = 1). Samples were collected at the indicated times after infection and subjected to western blot analysis or plaque assay. (**A**) Western blot analysis of LC3 and viral NS3 expression inhibited with Wort at 14 and 18 hpi. GAPDH was used as a protein loading control; (**B**) extracellular virus yields were determined by plaque assay on Vero cells and expressed as pfu/mL; (**C**) western blot analysis of LC3 and viral NS3 expression inhibited with Wort at the concentrations of 100, 300, and 500 nM at 18 hpi. (**D**) Determination of the extracellular virus yields (pfu/mL) at 18 hpi after treatment with Wort at different concentrations; (**E**) western blot analysis of LC3 and viral NS3 expression inhibited with CQ at 18 and 24 hpi. GAPDH was used as a protein loading control; (**F**) determination of the extracellular virus yields (pfu/mL); (**G**) western blot analysis of autophagy and viral NS3 expression inhibited with CQ at concentrations of 25, 50, and 75 µM at 24 hpi; (**H**) determination of the extracellular virus yields (pfu/mL) at 24 hpi after treatment with CQ at different concentrations. Data are presented as means ± SDs from three independent experiments. Significance is analyzed with two-tailed Student’s t test compared to the control group. * *p* < 0.05; ** *p* < 0.01; *** *p* < 0.001.

To analyze the effect of autophagy on ZIKV production in HUVEC, autophagic inhibitors, wortmannin (Wort) and chloroquine (CQ), were employed to block the autophagic response. Wort inhibits autophagy in the early stages by inhibiting the phosphatidylinositol3-kinase (PI3K) pathway. CQ acts as an autophagic inhibitor during the late stages, because it causes accumulation of sequestered material in either autophagosomes or autolysosomes [34]. As shown in Figure 3A,B, the conversion of LC3-I to LC3-II was reduced in Wort-treated HUVEC when compared to the control cells. Additionally, titers of viral progeny were noticeably reduced in Wort-treated cells, with a ~1.70-fold reduction occurring at both 14 and 18 hpi. Surprisingly, the expression of viral NS3 protein was not correspondingly decreased (Figure 3A). CQ treatment significantly increased the conversion of LC3-I to LC3-II in ZIKV-infected cells, and the expression of viral NS3 protein at 18 and 24 hpi was decreased. The yields of extracellular virus were decreased 6.51-fold at 18 hpi and 3.68-fold at 24 hpi (Figure 3E,F). Furthermore, both Wort and CQ treatment reduced the extracellular yields of ZIKV progeny (Figure 3D,H). The indicated concentrations of drugs did not affect the cell viability. These data from pharmacological experiments demonstrate that inhibition of autophagy, especially at late stages, reduces ZIKV production in HUVEC.

### 3.4. Knockdown of Autophagy-Related Genes (ATGs) Reduces ZIKV Production in HUVEC

**Figure 4:**
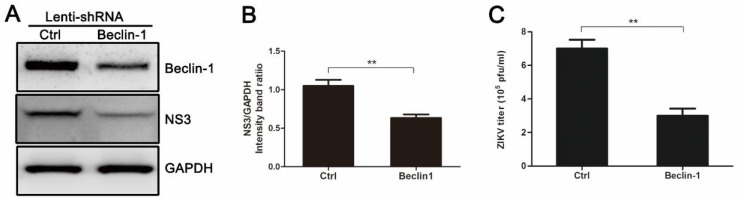
Gene silencing of Beclin-1 by pLenti-Beclin-1-shRNA (short hairpin RNA) inhibits ZIKV production. HUVEC were infected with empty lentivirus vector or pLenti-Beclin-1-shRNA. Forty-eight hours after lentivirus infection, cells were infected with ZIKV at an MOI of 1. Samples were collected at 24 h after ZIKV infection and subjected to Western blot analysis (**A**) or plaque assay (**C**). (**A**) Western blot analysis of Beclin-1 and viral NS3 expression. GAPDH was used as a protein loading control; (**B**) The ratios of NS3 to GAPDH were calculated and representative results are presented with graphs. Data are presented as means from three independent experiments. ** *p* < 0.01; (**C**) Extracellular virus yields were determined and expressed as pfu/mL. Data are presented as means ± SDs for triplicate assays. ** *p* < 0.01.

Beclin-1 is critical for the signaling pathways involved in autophagosome formation [15]. As shown in Figure 4A,B, HUVEC expressing Beclin-1-specific short hairpin RNA (shRNA) exhibited a decrease in endogenous Beclin-1 protein and viral NS3 protein when compared with control cells infected with an empty lentivirus vector. The reduced level of Beclin-1 protein resulted in a 2.33-fold decrease in extracellular virus yields (Figure 4C). These results suggest that autophagy machinery is required for effective infection of ZIKV, which agrees with the results of pharmacological experiments.

### 3.5. Rapamycin Does Not Promote ZIKV Production in HUVEC

**Figure 5:**
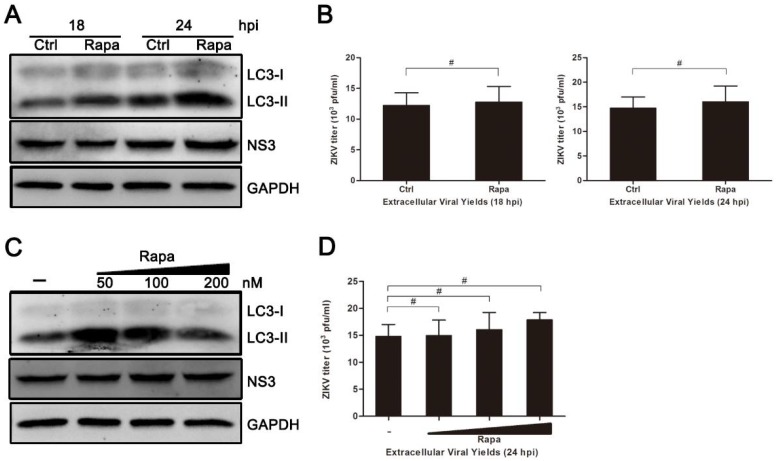
Treatment of rapamycin has no effect on ZIKV infection in HUVEC. (**A**) HUVEC were pretreated with rapamycin (Rapa) (100 nM) or DMSO (Ctrl) in complete medium and then processed as described for Figure 4A; (**B**) At 18 and 24 hpi, extracellular virus yields were determined by plaque assay on Vero cells and expressed as pfu/mL; (**C**) HUVEC were pretreated with Rapa or DMSO and then processed as described for panel A, with drug concentrations of 50, 100, 200, and 500 nM; (**D**) Extracellular virus yields at 24 hpi were determined and expressed as pfu/mL. Data are presented as means ± SDs from three independent experiments. Significance was analyzed with two-tailed Student’s t test and compared to the control group. # *p* > 0.05.

Inhibition of autophagy by either drugs or Beclin-1-specific shRNA reduced ZIKV production in HUVEC. To verify whether the induction of autophagy could enhance the production of ZIKV in infected cells, the use of rapamycin, an autophagy inducer, was employed. As shown in Figure 5, although the conversion of LC3-I to LC3-II appeared to increase slightly at 18 hpi, there was no significant change in both the expression of viral NS3 protein and extracellular yields of viral progeny observed during rapamycin treatment (Figure 5A–D).

## 4. Discussion

Autophagy is a common strategy for cell defense after a viral infection, but it is also reported that cellular autophagy can also be utilized by some viruses to benefit their own life cycle in host cells. For example, it is used to promote the replication of dengue virus, hepatitis C virus, Newcastle disease virus (NDV), coxsackievirus, and poliovirus [19,20,21,29,35]. In this study, we demonstrated that autophagy is also triggered in HUVEC after being infected with ZIKV.

The occurrence and degree of autophagy are usually evaluated by the observation of LC3 fluorescent spot formation, western blot analysis of LC3 conversion, and the degradation of p62/SQSTM1 [33,36]. Our data demonstrated an increase in the amount and distribution of LC3 fluorescent spots in ZIKV-infected HUVEC by confocal microscopy, indicating autophagy formation. The ratio of LC3-II/GAPDH band density increased at different time points (18, 24, and 36 h) after infections with different MOIs of ZIKV (Figure 1). This indicates that the conversion of LC3-I to lipidated LC3-II increases with increasing infection duration. In addition, the expression level of p62 was reduced starting at 12 h after ZIKV infection. GFP signals are susceptible to acidic conditions, but RFP is relatively stable [32]. Thus, yellow punctate dots (both red and green) indicate immature autophagosomes, and red dots indicate mature autolysosomes after fusion with lysosome. Increased autophagy was indicated after ZIKV infection by double-labeled fluorescence of LC3 (Figure 2). These observations suggest that ZIKV infection likely induces complete autophagy in HUVEC. However, whether ZIKV infection induces autophagy in other cell lines needs to be further investigated.

To further determine the role of autophagy in ZIKV infection, we investigated the effects of autophagy on viral replication and progeny virus production. By suppressing autophagy with wortmannin or chloroquine, we found a reduction in the yields of progeny virus. Additionally, we found that chloroquine treatment also reduced the synthesis of viral NS3 protein (Figure 3). In the case of Wort treatment, the expression of viral NS3 protein was not decreased significantly. It suggests that progeny virus formation is affected, rather than viral replication, upon the Wort treatment. However, Wort is not a specific inhibitor of the autophagosome, because it inhibits both class I and III PI3K [34,37]. Thus, we next assessed the effect of endogenous Beclin-1 on viral replication and production. As a core part of the class III PI3K (PI3K-III) lipid kinase complex, Beclin-1 is a key autophagic gene that plays a critical role in membrane trafficking and restructuring involved in cellular autophagy. As expected, knockdown of Beclin-1 significantly reduced both the expression of viral NS3 protein and the progeny virus titer when compared to the control (Figure 4). These results indicate that significant changes in ZIKV production result from the manipulation of host autophagy pathways by drug compounds or ATGs-specific RNA interference. They also suggest that ZIKV partially adopts the action of Beclin-1, which is required for the protective functions of autophagy, to benefit their own replication.

Rapamycin acts as an inhibitor of mTOR and usually induces cellular autophagy. However, the inhibition of mTOR activity is incomplete and varies by cell type. In some cases, cellular mTOR signaling is necessary for the replication and survival of several viruses, including poxvirus, herpesvirus, hepatitis C virus (HCV), and Chikungunya virus [38,39,40,41]. Although the conversion of LC3-I to LC3-II increased following rapamycin treatment in infected HUVEC, viral NS3 protein expression and the progeny virus titer were not increased significantly when compared with the control group without rapamycin pretreatment (Figure 5). These results are in agreement with previous studies performed in HCV and Andes virus (ANDV). Their results showed that rapamycin, or its analogue temsirolimus, inhibited HCV and ANDV replication in Huh-7.5 cells and primary human microvascular endothelial cells, respectively [42,43]. Treatment with rapamycin led to a reduction in the expression of several important viral proteins, and it even reduced virion release but not virus entry. In the case of ZIKV, whether mTORC1 signaling is also required for its efficient replication needs to be further studied.

The process of autophagosome formation is usually controlled by multiple signaling pathways, including the class III PI3K/Beclin-1 pathway mentioned above and the class I PI3K/Akt/mTOR pathway [14,44]. As a serine/threonine protein kinase, mTOR is one of the key regulators of the class I PI3K pathway, and it has a negative effect on the formation of autophagy [45,46]. Class I PI3K is a traditional upstream activator of mTOR. A previous study reported that ZIKV NS4A and NS4B proteins caused human fetal neural stem cells (fNSCs) nerve damage and induced autophagy by inhibiting the host Akt-mTOR signal transduction. This indicates that autophagy induced by ZIKV infection is regulated by the class I PI3K/Akt/mTOR pathway [23]. In the current study, rapamycin, an inhibitor of mTOR, triggered cellular autophagy, but it did not significantly promote viral protein expression and progeny virion production. This suggests that other regulators or mechanisms are involved in the ZIKV-infected HUVEC. Considering the role of mTOR signaling in HCV and ANDV replication, our data suggest that both the class I PI3K/Akt/mTOR pathway and the class III PI3K/Beclin-1 pathway are likely to be involved in the regulation of autophagy triggering in ZIKV-infected HUVEC. Different mechanisms of autophagy triggering in various cell types cannot be excluded, and the molecular mechanism of ZIKV pathogenesis in different cell types requires further clarification.

In conclusion, our data suggest that ZIKV infection can trigger autophagy in HUVEC, and it is likely to exploit host cell autophagy to promote the generation of its progeny virus, as seen in other members of the flavivirus family.
